# The Short- and Long-Term Outcome Priorities of a Western Australian Adult Burn Population

**DOI:** 10.1093/jbcr/irad175

**Published:** 2023-11-05

**Authors:** Inge Spronk, Fiona M Wood, Mark W Fear, Corine A Lansdorp, Dale W Edgar

**Affiliations:** Department of Public Health, Erasmus MC University Medical Centre Rotterdam, Rotterdam, 3000CA, The Netherlands; Association of Dutch Burn Centres, Maasstad Hospital, Rotterdam, 3007AC, The Netherlands; Dutch Burns Foundation, Beverwijk, 1941AJ, The Netherlands; State Adult Burn Unit, Fiona Stanley Hospital, South Metropolitan Health Service, Murdoch, WA 6150, Australia; Burn Injury Research Unit, School of Biomedical Sciences, University of Western Australia, Crawley, WA 6009, Australia; Fiona Wood Foundation, Murdoch, WA 6150, Australia; Burn Injury Research Unit, School of Biomedical Sciences, University of Western Australia, Crawley, WA 6009, Australia; Fiona Wood Foundation, Murdoch, WA 6150, Australia; Department Plastic, Reconstructive and Hand Surgery, Amsterdam UMC location Vrije Universiteit Amsterdam, Amsterdam, 1007MB, The Netherlands; State Adult Burn Unit, Fiona Stanley Hospital, South Metropolitan Health Service, Murdoch, WA 6150, Australia; Burn Injury Research Unit, School of Biomedical Sciences, University of Western Australia, Crawley, WA 6009, Australia; Fiona Wood Foundation, Murdoch, WA 6150, Australia; Institute for Health Research, Burn Injury Research Node, The University of Notre Dame Australia, Fremantle, WA 6959, Australia; Safety and Quality Unit, Armadale Kalamunda Group Health Service, East Metropolitan Health Service, Mt Nasura, WA 6992, Australia

**Keywords:** burns, outcomes, patient priorities

## Abstract

To optimize patient recovery, understanding which outcomes are most important to burn patients is key. However, research to determine what outcomes are patient priorities is limited. Therefore, we assessed what outcomes are most important to Western Australian burn patients, separately in the short-term (<6 months) and long-term (6-24 months) after injury. Adult patients who had a burn injury 3-36 months ago completed a survey, rating the importance of 36 short- and long-term outcomes. The survey items were ranked according to the number of patients reporting the outcome as “very important.” Results were compared between subgroups based on age, gender, burn size, and number of surgeries. Ninety-three patients were included. In the short-term, “not having a wound infection” (87.1%), “good wound healing” (83.9%), and “walking or moving around” (74.7%) were the most important outcomes. “Lifting or moving something” (67.6%), “walking or moving around” (66.2%), and “being independent” (66.2%) were reported as most important in the long-term. Scar-related outcomes were more important to females and to patients with multiple surgeries; mental health outcomes were priorities for females and patients with major burns; walking and moving around to males and older patients; and social and financial outcomes were rated highly by patients with major burns and multiple surgeries. In conclusion, the most important outcomes were consistent across time periods, indicating the importance of core outcomes in longitudinal follow-up. The wide range of priority outcomes and differences between subgroups underlines the need for multidisciplinary care and a patient-centered approach to support patients.

## INTRODUCTION

Surviving a burn injury has a tremendous long-term impact on a patient’s life.^1,2^ Patients need specialized burn care, often including surgery, skin grafting, and hospital admission. Rehabilitation and returning to daily life may take a long time.^3,4^ Patients live with scarring, functional and mental health problems, which negatively impact their quality of life.^5,6^

An important goal of specialized burn care is to achieve the best patient-focused health outcomes and optimizing value across all domains. A strategy to support this is value-based healthcare (VBHC), which aims to achieve the best patient-relevant health outcomes at the lowest costs per patient.^7^ VBHC has not yet been systematically implemented in burn care, and is proposed to deliver a major positive impact as a driver for opportunities to reduce variation in practice for the broad spectrum of patient characteristics, the multitude of primarily empirical treatment strategies currently used, the lack of gold or evidence-based standards and the high relative costs of interventions.^8–11^ Results achieved by implementing VBHC in other contexts include improved patient value and satisfaction, decreased hospital stay, fewer complications, and enhanced quality of life.^12–14^ These outcomes, if achieved through the burn VBHC context, would clearly constitute a worthwhile investment for patients and burn care services.

An essential component of VBHC is measuring and evaluating outcomes that are most important to patients.^9,10^ These outcomes should include assessment of short- and long-term health consequences.^15^ A substantial body of research exists presenting frameworks for outcome assessment after burn injuries, arising from reviews across the total spectrum of consequences after burns including physical, psychological, and social functioning, and also, specific health domains, such as contractures, scarring, and return to work.^1,3,16–19^ Further, within burn care, there is a growing interest in and use of patient-reported outcomes (PROs).^1,20–22^ PROs record aspects of health as directly assessed by a patient without interpretation of third parties,^23,24^ and provide a view of the individual’s perspective on their own health that goes beyond any physiological measure. This is a central premise of VBHC. However, (patient-reported) outcomes are usually selected by researchers and/or healthcare providers with limited consultation or involvement of patients.

Several studies have explored important outcomes in the field of burns, however, a specific examination of outcomes that matter most to patients themselves has been somewhat lacking. Notably, many of the existing studies encompass not only the perspectives of patients but also include the views of healthcare professionals and researchers.^25^ Additionally, the predominant nature of studies exclusively focusing on burn patients has leaned towards qualitative rather than quantitative methodologies. These studies primarily aimed to uncover the domains that bear significance for patients, albeit often without the concurrent task of ranking the relative importance of specific outcomes in comparison to one another.^26^ A recent systematic review conducted an exhaustive assessment of all qualitative studies involving adult burn patients, resulting in the establishment of a comprehensive framework encompassing significant outcomes.^27^ These qualitative research foundations hold considerable significance. However, the majority of the qualitative studies included in the review included small samples of patients. Furthermore, the framework proposed primarily addresses outcome domains rather than specific outcome constructs.^27^ For instance, when considering the domain of scarring and scar characteristics, the framework does not provide precise guidance on the aspects that a clinician should assess or measure. Outcomes that matter most according to patients are key to defining, monitoring, and enhancing patient value. These outcomes should be used to evaluate and improve burn care. While previous studies have indicated that a wide spectrum of outcome domains bear significance for adult burn patients, there remains a notable gap in our understanding regarding which specific outcomes are of the greatest importance to patients alone. Recently, a Dutch study showed that not having pain was very important during admission and that good wound healing, being independent, and taking care of yourself were the most important outcomes for patients during the recovery of burns.^28^ It is, however, unknown whether these results are generalizable to other countries, local jurisdictions, and cultures. Our aim was therefore to assess what outcomes are most important to Western Australian patients, separately in the short-term (<6 months postburn) and long-term (6-24 months postburn) after burn injury and compare results to the previous Dutch study.

## METHODS

The Child and Adolescent Health Service Human Research Ethics Committee reviewed and approved cross-sectional survey studies for both adults and children (RGS5644). The results of the study on children and their carers will be presented separately. The South Metropolitan Health Service provided Site Governance approval for the adults’ survey study. This study was performed in line with the principles of the Declaration of Helsinki, and followed the “Strengthening the Reporting of Observational studies in Epidemiology” (STROBE) guidelines.^29^ Participants provided informed e-consent before completing the survey.

### Study population and procedure

Adult burn patients (≥18 years old) who had a burn injury 3-36 months earlier and were treated at the adult burn unit of the Fiona Stanley Hospital in Perth were extracted from the hospital registry in March 2023. Patients were not eligible if the individual was under the care of the Mental Health Act 2014. All eligible patients who had pre-registered their email address were selected and invited to participate in this study. Identified patients received an email invitation, accompanied by a patient information sheet and a link to the online survey. After informed e-consent was received, each participant proceeded to anonymously answer the survey questions. If patients did not respond to the survey within 3 weeks, they received a reminder invitation, and a second reminder was sent 6 weeks after the initial contact.

### Survey

The survey was an adapted version of the survey on “What outcomes matter most to burn patients”^28^ (Supplementary Appendix 1). To ensure alignment with the Australian context and culture, the English version of the survey underwent a thorough evaluation by 6 Australian healthcare providers and patient advocates resulting in minor language and style adjustments. The registry software REDCap was used to deliver and capture e-consent and responses.^30^ It included items on patient and clinical characteristics (6 questions), the importance of short-term outcomes (<6 months postburn) (36 questions), and the importance of long-term outcomes (6-24 months postburn) (34 questions). If patients were <6 months postburn, the questions on long-term outcomes were hidden, using branching logic. Patient and clinical characteristics captured, included age, gender, percentage total body surface area (%TBSA) burned, burn unit admission, length of stay (if applicable), number of surgeries (0/1/>1), and time since injury in months. The short- and long-term items were almost identical for both time categories; only 2 outcomes (wound healing and wound infection) were not included in the long-term outcomes. The outcomes were based on the International Classification of Functioning Disability and Health (ICF) framework and divided into 5 domains: physical recovery, scar(s), emotions, daily activities, and roles and relationships.^31,32^ Each item had 4 answer options: “not important,” ‘moderately important, “very important,” and “not applicable/I don’t know.”

### Statistical analyses

Patient responses were included when they completed at least one item on the importance of outcomes. Descriptive analyses were used to describe patient and clinical characteristics and to uncover what outcomes were considered most important to patients, separately in the short-term and long-term recovery from burns. Continuous data were reported as mean (SD) if normally distributed, or as median (IQR) if not-normally distributed. Categorical data were reported as numbers (percentage). Outcomes were ranked on the number of patients scoring an outcome as “very important.” Results were studied for the total sample, and compared between subgroups based on gender (males vs. females), age (≤45 vs >45 years old),^33^ burn size ≤15% vs >15% TBSA burned),^34^ and surgery for burns (no surgery vs 1 surgery vs ≥2 surgeries) using chi-square tests, or Fisher’s exact tests in case of small numbers (<5). A *P*-value of <.05 was considered statistically significant. Python 3.11 was used for the analyses.

## RESULTS

A total of 447 patients were contacted from the hospital registry, of whom 93 (20.8%) completed the survey and were included. The median age was 49.0 (IQR: 32.0-57.0) years old, and slightly more than half of the participants were female (51.6%) (Table 1). Participants’ median %TBSA burned was 5.0% (IQR: 2.0%-15.0%) and 80.6% of them were admitted for their burn injury. Most participants (75.3%) had surgery for their burns. The median time after injury was 18.0 months (IQR: 7.0-30.0).

**Table 1.** Patient Characteristics

Variable	Total sample (*n* = 93)
Sex: male, *n* (%)	45 (48.4%)
Age, median (IQR)	49.0 (32.0-57.0)
≤45 years old	43 (46.2%)
>45 years old	50 (53.8%)
%TBSA burned, median (IQR)	5.0 (2.0-15.0)
≤15% TBSA burned	72 (77.4%)
>15% TBSA burned	21 (22.6%)
Hospital admission, *n* (%)	75 (80.6%)
Length of hospital stay (days), median (IQR)	5.0 (1.0-10.0)
Surgery, *n* (%)	
No surgery	23 (24.7%)
One surgery	48 (51.6%)
More than one surgery	22 (23.7%)
Time since burn (months), median (IQR)	18.0 (7.0-30.0)

### Priority of outcomes

#### Short-term

**Figure 1.**
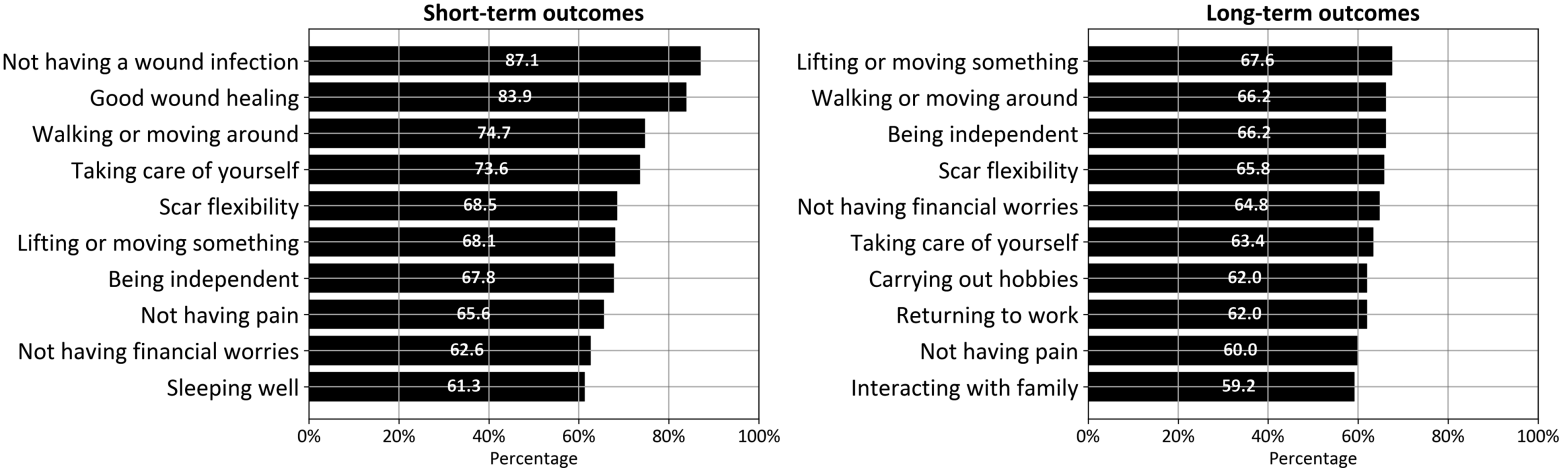
Top-10 Most Important Short- and Long-Term Outcomes.

All 93 patients completed the items on short-term outcomes. “Not having a wound infection” was reported to be very important by most patients (87.1%), followed by “good wound healing” (83.9%), and “walking or moving around” (74.7%) (Figure 1). Other outcomes that were very important to more than two-thirds of the participants included “taking care of yourself” (73.6%), “scar flexibility” (68.5%), “lifting or moving something” (68.1%), “being independent” (67.8%). The three least important outcomes in the short-term included “interacting with your boss” (16.7%), “physically being able to have sex” (25.6%), and “not having nightmares” (29.3%) (Appendix 2).

#### Long-term

Seventy-nine of the 93 patients (84.9%) were ≥6 months postburn and completed the items on long-term outcomes. The most important outcomes were similar in the short- and long-term (Figure 1), however, the proportion of patients finding an outcome “very important” reduced with increasing time after burn. “Lifting or moving something” (67.6%), “walking or moving around” (66.2%), and “being independent” (66.2%) were the most frequent of the long-term responses that were considered “very important.” “Carrying out hobbies” (62.0% vs 53.8%), “returning to work” (62.0% vs 53.8%), and "not having financial worries" (62.6% vs 64.8%) increased in importance. The least important outcomes included “interacting with your boss” (25.4%), “not thinking back to the incident” (27.4%) and ‘not feeling guilty or ashamed (31.9%) (Appendix 3).

### Priority of outcomes for males vs females

#### Short-term

Important outcomes were similar between males and females (Table 2). “Walking or moving around” was more important to males (88.9% vs 60.9%; *P* = .021), whereas “the look/appearance of the scar(s)” was more important to females (55.3% vs 15.6%, *P* < .001). Also, “having self-confidence,” “not being anxious,” “not feeling guilty or ashamed,” and “your appearance” were all more important to females (*P* < .05).

**Table 2.** Top-10 Most Important Short-Term and Long-Term Outcomes for Males and Females

Males		Females	
<6 months postburn	*n* (%)	<6 months postburn	*n* (%)
Not having a wound infection	41 (91.1%)	Not having a wound infection	40 (83.3%)
Walking or moving around	40 (88.9%)	Good wound healing	39 (81.2%)
Good wound healing	39 (86.7%)	Scar flexibility	34 (72.3%)
Taking care of yourself	34 (75.6%)	Taking care of yourself	33 (71.7%)
Lifting or moving something	31 (68.9%)	Being independent	32 (71.1%)
Scar flexibility	29 (64.4%)	Having self-confidence	33 (70.2%)
Being independent	29 (64.4%)	Not having pain	33 (68.8%)
Sleeping well	29 (64.4%)	Lifting or moving something	31 (67.4%)
Not having financial worries	28 (62.2%)	Not having financial worries	29 (63.0%)
Not having pain	28 (62.2%)	Walking or moving around	28 (60.9%)

6-24 months postburn	*n* (%)	6-24 months postburn	*n* (%)
Walking or moving around	29 (80.6%)	Scar flexibility	26 (72.2%)
Lifting or moving something	28 (77.8%)	Being able to cope with heat	23 (63.9%)
Not having financial worries	27 (75.0%)	Not having itching	24 (63.2%)
Taking care of yourself	26 (72.2%)	Being independent	22 (62.9%)
Being independent	25 (69.4%)	Having energy	23 (60.5%)
Returning to work	24 (66.7%)	Carrying out hobbies	21 (60.0%)
Carrying out hobbies	23 (63.9%)	Interacting with family	21 (60.0%)
Not having pain	23 (62.2%)	Feeling happy or cheerful	21 (58.3%)
Sleeping well	22 (59.5%)	Having self-confidence	21 (58.3%)
Scar flexibility	22 (59.5%)	Look/appearance of the scar(s)	21 (58.3%)

#### Long-term

The most important outcomes differed between males and females. For males, the top-10 primarily included physical and social outcomes, with “walking and moving around” (80.6%), “lifting or moving something” (77.8%), and “not having financial worries” (75.0%) being the top 3. Whereas for females, the top-3 included “scar flexibility” (72.2%), “being able to cope with heat” (63.9%), and “not having itching” (63.2%). However, the only statistically significant difference was “the look/appearance of the scar(s)” which was more important to females (*P* < .001).

### Priority of outcomes for younger and older patients

#### Short-term

**Figure 2.**
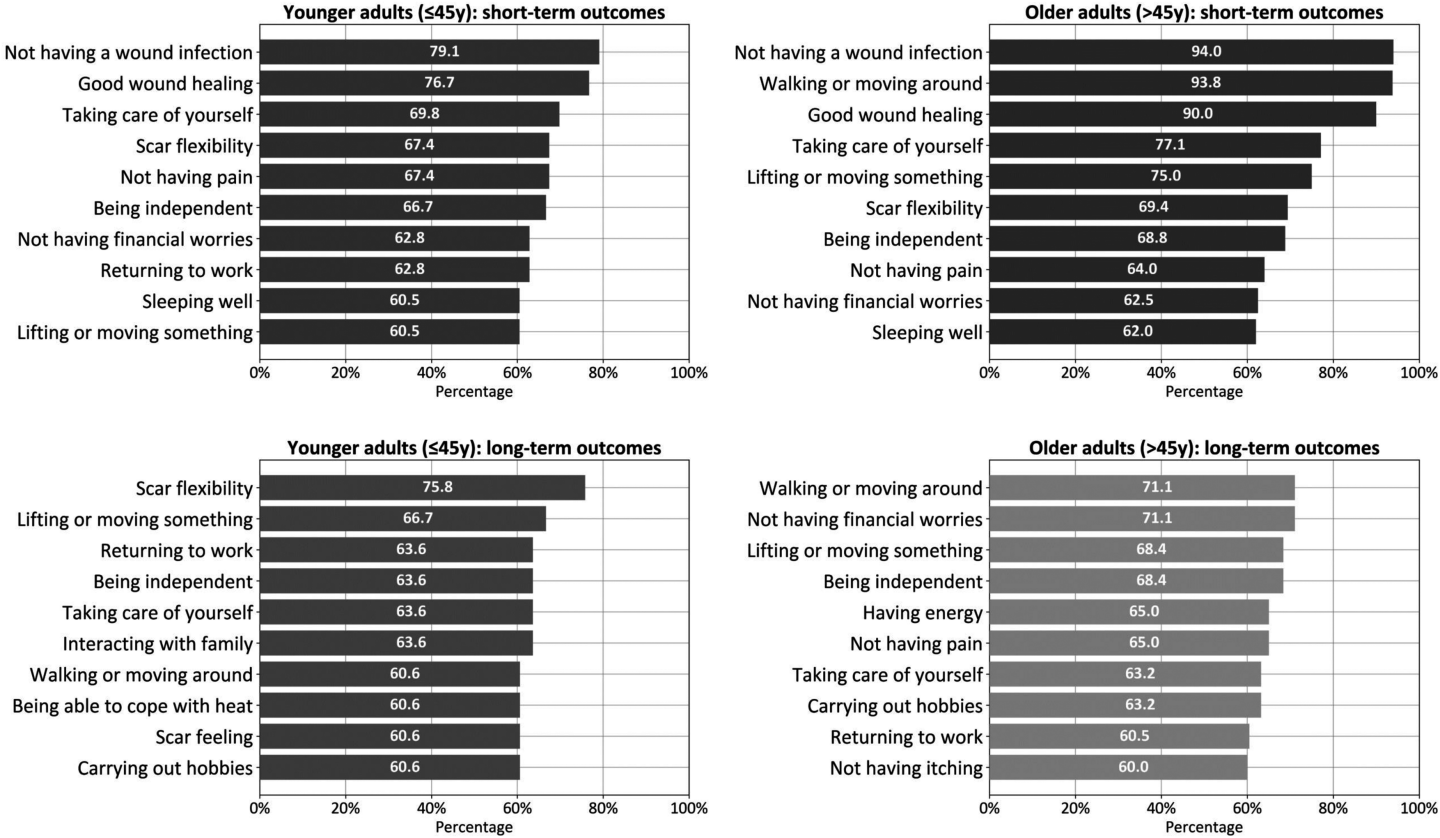
Top-10 Most Important Short- and Long-Term Outcomes for Younger (≤45 y Old) and Older Patients (>45 y Old).

The most important outcomes were similar between younger (≤45 years old) and older patients (>45 years old), except for “walking or moving around,” which was more important to older patients (53.5% vs 93.8%; *P* < .001); and “interacting with friends/colleagues” which was more important to younger patients (57.1% vs 33.3%; *P* = .041) (Figure 2).

#### Long-term

More differences were evident in the long-term outcome ratings. “Scar flexibility” was most important to young patients (75.8% vs 57.5%), whereas “not having financial worries” was more important to older patients (71.1% vs 57.6%). The only significant difference was the importance of “interacting with family,” which was more important to younger patients (63.6% vs 55.3%; *P* = .023).

### Priority of outcomes for patients with minor and major burns

#### Short-term

Patients with minor burns (≤15% TBSA burned) and major burns (>15% TBSA burned) indicated the same outcomes were very important (Table 3). Four mental health outcomes, “not being anxious” (*P* = .010), “not feeling depressed” (*P* = .026), “not having nightmares” (*P* = .001), and “not thinking back to the incident” (*P* = .011), were statistically more important to patients with major burns.

**Table 3.** Top-10 Most Important Short-Term and Long-Term Outcomes for Patients With Minor Burns (≤15% TBSA Burned) and Major Burns (>15% TBSA Burned)

Patients ≤15% TBSA burned		Patients >15% TBSA burned	
<6 months postburn	*n* (%)	<6 months postburn	*n* (%)
Not having a wound infection	61 (84.7%)	Good wound healing	20 (95.2%)
Good wound healing	58 (80.6%)	Not having a wound infection	20 (95.2%)
Taking care of yourself	49 (70.0%)	Walking or moving around	19 (90.5%)
Walking or moving around	49 (70.0%)	Scar flexibility	18 (85.7%)
Lifting or moving something	47 (67.1%)	Taking care of yourself	18 (85.7%)
Being independent	45 (65.2%)	Not having pain	17 (81.0%)
Scar flexibility	45 (63.4%)	Being independent	16 (76.2%)
Not having pain	44 (61.1%)	Sleeping well	16 (76.2%)
Not having financial worries	42 (60.0%)	Not feeling depressed	16 (76.2%)
Sleeping well	41 (56.9%)	Not having financial worries	15 (71.4%)

6-24 months postburn	*n* (%)	6-24 months postburn	*n* (%)
Being independent	33 (62.3%)	Returning to work	18 (100.0%)
Taking care of yourself	33 (62.3%)	Scar flexibility	17 (89.5%)
Lifting or moving something	32 (60.4%)	Lifting or moving something	16 (88.9%)
Walking or moving around	32 (60.4%)	Having energy	15 (78.9%)
Not having financial worries	31 (58.5%)	Not having financial worries	15 (83.3%)
Carrying out hobbies	31 (58.5%)	Walking or moving around	15 (83.3%)
Scar flexibility	31 (57.4%)	Not having pain	14 (73.7%)
Not having pain	31 (55.4%)	Not having itching	14 (73.7%)
Not having itching	30 (53.6%)	Interacting with family	14 (77.8%)
Interacting with family	28 (52.8%)	Being independent	14 (77.8%)

#### Long-term

In contrast to the short-term, the importance of long-term outcomes differed between the 2 groups. The most important outcome for patients with minor burns was “taking care of yourself,” which did not appear in the top 10 of patients with major burns. In contrast, “returning to work” was found very important by all patients with major burns, but only by half of the patients with minor burns (49.1%; *P* = .002). In addition, 6 cognitive and mental health outcomes, including “being able to think well” (45.3% vs 73.7%, *P* = .010) were significantly more important to patients with major burns (*P* < .05).

### Priority of outcomes for subgroups based on number of surgeries

#### Short-term

Two significant differences were present among the 3 subgroups; “fine hand motor skills” (77.3%) and “interacting with partner” (59.1%) were more important to patients with ≥2 surgeries compared to patients with no surgery (43.5% and 26.1%, respectively) and with one surgery (45.7% and 40.0%, respectively; *P* = .014-.027) (Appendix 4).

#### Long-term

More variation in priorities was evident in the long-term. “Scar flexibility” was most important to patients without surgery (52.6%) and to those with ≥2 surgeries (90.0%), however, it was only the 9th important outcome for patients with one surgery (58.8%, *P* = .029). Interacting with others, including partners, family, and boss, was statistically more important to patients with ≥2 surgeries compared to the other subgroups (*P* = .009-.045), as was “not feeling depressed” (*P* = .035) and “physically being able to have sex” (*P* = .029).

## DISCUSSION

This study confirmed the most important short-term outcomes for patients were related to wound healing, physical functioning, and scars. Mental health and social outcomes were considered less important in the early timeframe after their burn. The most important long-term outcomes were similar to those in the short-term, indicating the importance of longitudinal follow-up to monitor the restoration of patient-centered quality of life across multiple domains. Social outcomes increased in importance in the long-term for the total sample.

Our findings are in line with that of an earlier study that investigated the outcomes that were important to burn patients during scar management; 8 core domains were defined of which most are covered by the most important outcomes uncovered in our study.^35^ Comparing our results to the Dutch study (sample [*n*=140]: mean age = 53 years; 62% male; mean TBSA burned = 9%) on what outcomes matter most, the proportion of patients finding the top-10 outcomes very important were comparable, as were the highest priority short- and long-term outcomes.^28^ Interestingly, lower-ranked top-10 important outcomes differed between both samples. In general, Dutch patients found general well-being, including trusting their body, and mental health and cognitive outcomes, including having self-confidence and being able to think well, more important, whereas Western Australian patients found scar-related, pain, and financial outcomes, including returning to work, more important. In addition to cultural differences, these disparate results may be partly explained by differences in the model of care, health and income insurances; and, patient characteristics in both samples. In Western Australia, the Statewide model of burn care is geared to early acute wound closure, with access to surgery within 3-5 days of injury, whereas in the Netherlands the main approach is generally considered conservative, resulting in longer hospital stays.^36,37^ A substantially larger proportion of Western Australian patients underwent surgery (75% vs 45%), which might explain the differences in the importance of scar-related outcomes and pain. The differences in the importance of mental health outcomes might also be partly explained by the differences in length of hospital admission, which was considerably longer in the Dutch sample. Earlier studies showed that a prolonged hospital stay is associated with more mental health problems.^38–40^ The differences in the importance of financial worries and returning to work seem not to be explained by gender roles as a larger proportion of the Dutch study was male (62% vs 48%). While Western society is changing, males remain more likely to be responsible for the family income than females^41,42^ and consequently may feel these outcomes are more important. However, the Western Australian sample included a higher proportion of females but found financial worries and returning to work more important. The social support system in both countries is very different and could explain the different focus of patients on income and employment between the 2 studies. In the Netherlands, employers are obliged to pay at least 70% of the wage for up to 2 years if an individual is not able to work due to sickness or injury and help the employee in their return-to-work process, including job adaptations, retraining, and/or getting a new job.^43^ In contrast in Australia, whilst employers are obligated to provide leave entitlements for short-term incapacity, many people experience a large drop in their income if they are unable to work for a prolonged period and do not have private life insurance.^44^ It is thus not surprising that Western Australian patients find having no financial worries and returning to work more important than Dutch patients. The overlap of the most important outcomes in both countries concurs with a global core set, with a limited number of outcomes important for burn care and research.^25^ However, the differences in the other top-10 outcomes highlight the impact of cultural, care, and social system differences and indicate the need for country-specific adaptations to such a core set.

Commonalities were evident in the most important outcomes for the different subgroups studied, especially in the short-term. However, some differences were evident. Mental health outcomes were more important to females and to patients with major burns, in line with existing evidence about the prevalence of these consequences in these subgroups.^38–40,45^ Walking was more important to males and older patients, whereas interacting with friends was more important to younger patients. Fine motor skills and interacting with partners were more important to patients with ≥2 surgeries. Scar outcomes were most important to females and to patients who had multiple surgeries, both in the short- and long-term. Generally, in social culture, more attention is paid to females’ appearance compared to males’ appearance, which might at least in part explain this difference. An earlier study showed that females’ opinion of their scars was worse than males, even if their own more objective evaluation was not.^46^ However, some evidence suggests that females have an increased risk of a raised scar in particular in response to burns, due to more maladaptive wound healing responses thought to be linked to differences in the immune and hormone system.^47^ However, other studies on scar quality and hypertrophic scars have not shown sex differences.^36,48–50^

In the longer term, differences in the importance of outcomes were mainly seen between patients with non-severe and severe burn injuries, either defined by %TBSA burned or a number of surgeries. Being independent, having no financial worries and returning to work were very important to patients with severe burn injuries, but not to those with non-severe burns, possibly because these topics are not a concern to them in the longer term. Also, interacting with others and mental health outcomes were more important to patients with severe burns, showing the large impact of larger burns. The wide range of important outcomes underlines the need for multidisciplinary care to support burn patients, especially those with severe burns. In addition, the differences observed between subgroups indicate the importance of a patient-centered approach.

The most important outcomes identified in this study are recommended for use to assess and evaluate Western Australian burn care and recovery over time. Hereby, outcomes of different treatment strategies can be quantified and compared, supporting care improvements and increasing patient value.^9,10,22^ In addition, insights from these patient-relevant outcomes can be applied and used to inform patients better on expected prognosis associated with different injuries and, or treatment options.^51^ Presenting the outcomes of importance in association with the potential impact of interventions to patients supports discussion with their healthcare provider as to what treatment best meets their needs, and values and promotes shared decision-making.^52–54^

This study included a number of strengths and limitations. This is the first study that asked burn patients about their opinion on an extensive set of outcomes in the Australian context. We used an adapted shortened version of the survey on “what outcomes matter most to burn patients” to reduce patient burden; the main part of the survey remained unchanged to be able to compare results. The distinction of time since burn was used to make sure that patients only completed questions that were relevant to them. Another strength was the sample size of the study, which enabled us to perform subgroup analyses. However, some subgroups had relatively small numbers and this limits the statistical power to uncover significant differences between subgroups. Nonetheless, significant differences were found, including important differences between subgroups. Due to the anonymous nature of the survey, we were unable to perform a non-response analysis and can therefore make no conclusion on whether the responders were representative of the total population. Another limitation was that the survey was only available online, which may have decreased the number of responses as an earlier study has shown that about one in 6 burn patients have difficulties with an online format.^28^

## CONCLUSION

This study showed the outcomes that are important to Western Australian burn patients in both the early and late phases of recovery from their burn injury. The most important outcomes were consistent across both time periods, indicating the importance of longitudinal follow-up. The wide range of important outcomes underlines the need for multidisciplinary care to support burn patients. Differences between subgroups such as sex and burn severity indicate the importance of a patient-centered approach, especially for the long-term evaluation of patient value and quality of burn care.

## Supplementary Material

irad175_suppl_Supplementary_Appendixs_S1-S4

## Notes

*Author Contributions:* Inge Spronk (Conceptualization [Equal]; Data curation [Equal]; Formal analysis [Lead]; Funding acquisition [Lead]; Investigation [Lead]; Methodology [Equal]; Project administration [Lead]; Resources [Equal]; Software [Lead]; Visualization [Lead]; Writing—original draft [Lead]), Fiona M Wood (Conceptualization [Equal]; Funding acquisition [Supporting]; Investigation [Supporting]; Methodology [Supporting]; Project administration [Supporting]; Resources [Supporting]; Supervision [Supporting]; Validation [Supporting]; Writing—review & editing [Supporting]), Mark W Fear (Conceptualization [Equal]; Investigation [Supporting]; Methodology [Supporting]; Project administration [Supporting]; Resources [Supporting]; Writing—review & editing [Supporting]), C A Lansdorp (Conceptualization [Equal]; Investigation [Supporting]; Methodology [Lead]; Validation [Equal]; Writing—review & editing [Equal]), and Dale W Edgar (Conceptualization [Equal]; Data curation [Supporting]; Formal analysis [Supporting]; Funding acquisition [Supporting]; Investigation [Equal]; Methodology [Equal]; Project administration [Lead]; Resources [Equal]; Software [Supporting]; Supervision [Lead]; Validation [Lead]; Visualization [Supporting]; Writing—review & editing [Lead])

*Funding*: This study was funded by the Dutch Burns Foundation.
