# Supplementary material for: The Short- and Long-Term Outcome Priorities of a Western Australian Adult Burn Population
Source: J Burn Care Res. 2023 Nov 5;45(2):451–8. doi: 10.1093/jbcr/irad175 (PMC10911694; doi:10.1093/jbcr/irad175)
Supplement: irad175_suppl_Supplementary_Appendixs_S1-S4 [file irad175_suppl_supplementary_appendixs_s1-s4.docx]

**Appendix 1. Survey development and pre-deployment testing**

The survey was developed by the Dutch Burn Care, Education & Research group [1]. The ensure coverage of all relevant domains, the International Classification of Functioning Disability and Health (ICF) framework was used in the survey development. Relevant items to be included in the survey were based on literature review and often used questionnaires in the field of burns and health [2-5]. Items were selected, and if needed redefined, in close collaboration with healthcare providers, patient and patient advocates, experience experts, and the Dutch Association of Burn Survivors.

The initial survey was critically reviewed by healthcare providers, researchers, patient advocates and experience experts from the three Dutch burn centres (n=22). This panel critically assessed the survey items, scrutinizing their suitability and relevance. Besides, they evaluated whether all important outcomes were covered by the survey items. Slight adaptations were made based on this evaluation. Following this, the survey was tested by 10 patients, experience experts and patient advocates. The final survey underwent translation into English. Two researchers independently translated the survey items, engaged in discussions with a bilingual individual, and collectively selected the most appropriate translations for the survey items. Subsequently, a separate researcher, uninvolved in the survey's initial development, conducted a backward translation. The backward translation was then compared to the original survey, revealing no significant discrepancies.

To ensure alignment with the Australian context and culture, the English version of the survey underwent a thorough evaluation by six Australian healthcare providers and patient advocates resulting in minor language and style adjustments. As a result, three survey items were slightly adjusted to better resonate with the Australian context and culture. Two items (‘not having a wound infection’ and ‘good wound healing’) were omitted from the original survey at the 6–24-month. This decision was made because these particular outcomes were deemed irrelevant within this specific time span. Additionally, in order to reduce the patient burden, the adapted version of the survey retained two of the original three time periods: short-term outcomes (<6 months postburn) (consisting of 36 questions) and long-term outcomes (6-24 months postburn) (comprising 34 questions). Notably, the original version also encompassed the timeframe 'during admission' [1].

**References**

[1] Spronk I, van Uden D, van Dammen L, et al. Outcomes that matter most to burn patients: a national multicentre survey study in the Netherlands. Accepted by Burns; 2023.

[2] Griffiths C, Guest E, White P, et al. A systematic review of patient-reported outcome measures used in adult burn research. J Burn Care Res. 2017;38:e521-e45.

[3] Spronk I, Legemate C, Oen I, et al. Health related quality of life in adults after burn injuries: a systematic review. PLoS One. 2018;13:e0197507.

[4] Kool MB, Geenen R, Egberts MR, et al. Patients’ perspectives on quality of life after burn. Burns. 2017.

[5] Young AE, Davies A, Bland S, et al. Systematic review of clinical outcome reporting in randomised controlled trials of burn care. BMJ open. 2019;9:e025135.

**Appendix 2. Importance of outcomes in the short-term (<6 months) recovery from burn injuries**

| **Outcome** | **Not important (n, %)** | **Moderately important (n, %)** | **Very important (n, %)** | **Not applicable (n, %)** |
| --- | --- | --- | --- | --- |
| Not having pain | 6 (6.5%) | 25 (26.9%) | 61 (65.6%) | 1 (1.1%) |
| Not having itching | 8 (8.6%) | 36 (38.7%) | 48 (51.6%) | 1 (1.1%) |
| Good wound healing | 1 (1.1%) | 12 (12.9%) | 78 (83.9%) | 2 (2.2%) |
| Not having a wound infection | 1 (1.1%) | 8 (8.6%) | 81 (87.1%) | 3 (3.2%) |
| Sleeping well | 6 (6.5%) | 26 (28.0%) | 57 (61.3%) | 4 (4.3%) |
| Having energy | 7 (7.5%) | 36 (38.7%) | 44 (47.3%) | 6 (6.5%) |
| Look/appearance of the scar(s) | 31 (33.7%) | 27 (29.3%) | 33 (35.9%) | 1 (1.1%) |
| Scar feeling | 13 (14.1%) | 35 (38.0%) | 43 (46.7%) | 1 (1.1%) |
| Scar flexibility | 4 (4.3%) | 24 (26.1%) | 63 (68.5%) | 1 (1.1%) |
| Being able to cope with heat | 8 (8.7%) | 39 (42.4%) | 44 (47.8%) | 1 (1.1%) |
| Not being anxious | 19 (20.7%) | 33 (35.9%) | 37 (40.2%) | 3 (3.3%) |
| Not having nightmares | 32 (34.8%) | 24 (26.1%) | 27 (29.3%) | 9 (9.8%) |
| Feeling happy or cheerful | 12 (13.0%) | 30 (32.6%) | 47 (51.1%) | 3 (3.3%) |
| Having self-confidence | 20 (21.7%) | 20 (21.7%) | 49 (53.3%) | 3 (3.3%) |
| Not thinking back to the incident | 27 (29.3%) | 24 (26.1%) | 36 (39.1%) | 5 (5.4%) |
| Not having stress | 16 (17.6%) | 27 (29.7%) | 45 (49.5%) | 3 (3.3%) |
| Not feeling depressed | 16 (17.6%) | 27 (29.7%) | 46 (50.5%) | 2 (2.2%) |
| Not feeling guilty or ashamed | 31 (34.1%) | 19 (20.9%) | 37 (40.7%) | 4 (4.4%) |
| Trusting your body | 17 (18.7%) | 27 (29.7%) | 42 (46.2%) | 5 (5.5%) |
| Being able to think well | 20 (22.0%) | 27 (29.7%) | 39 (42.9%) | 5 (5.5%) |
| Walking or moving around | 7 (7.7%) | 12 (13.2%) | 68 (74.7%) | 4 (4.4%) |
| Lifting or moving something | 10 (11.0%) | 16 (17.6%) | 62 (68.1%) | 3 (3.3%) |
| Fine hand motor skills | 15 (16.5%) | 13 (14.3%) | 48 (52.7%) | 15 (16.5%) |
| Taking care of yourself | 8 (8.8%) | 10 (11.0%) | 67 (73.6%) | 6 (6.6%) |
| Doing household chores | 13 (14.3%) | 29 (31.9%) | 42 (46.2%) | 7 (7.7%) |
| Carrying out hobbies | 14 (15.4%) | 26 (28.6%) | 49 (53.8%) | 2 (2.2%) |
| Returning to work | 13 (14.3%) | 21 (23.1%) | 49 (53.8%) | 8 (8.8%) |
| Not having financial worries | 14 (15.4%) | 14 (15.4%) | 57 (62.6%) | 6 (6.6%) |
| Being independent | 6 (6.7%) | 19 (21.1%) | 61 (67.8%) | 4 (4.4%) |
| Your appearance | 19 (21.1%) | 27 (30.0%) | 40 (44.4%) | 4 (4.4%) |
| Interacting with people/strangers | 25 (27.8%) | 33 (36.7%) | 27 (30.0%) | 5 (5.6%) |
| Interacting with friends/colleagues | 22 (24.4%) | 24 (26.7%) | 40 (44.4%) | 4 (4.4%) |
| Interacting with your boss | 34 (37.8%) | 23 (25.6%) | 15 (16.7%) | 18 (20.0%) |
| Interacting with family | 12 (13.3%) | 23 (25.6%) | 47 (52.2%) | 8 (8.9%) |
| Interacting with partner | 12 (13.3%) | 21 (23.3%) | 37 (41.1%) | 20 (22.2%) |
| Physically being able to have sex | 20 (22.2%) | 28 (31.1%) | 23 (25.6%) | 19 (21.1%) |

**Appendix 3. Importance of outcomes in the short-term (6-24 months) recovery from burn injuries**

| **Outcome** | **Not important (n, %)** | **Moderately important (n, %)** | **Very important (n, %)** | **Not applicable (n, %)** |
| --- | --- | --- | --- | --- |
| Not having pain | 10 (13.3%) | 14 (18.7%) | 45 (60.0%) | 6 (8.0%) |
| Not having itching | 8 (10.7%) | 16 (21.3%) | 44 (58.7%) | 7 (9.3%) |
| Sleeping well | 10 (13.3%) | 16 (21.3%) | 42 (56.0%) | 7 (9.3%) |
| Having energy | 10 (13.3%) | 15 (20.0%) | 44 (58.7%) | 6 (8.0%) |
| Look/appearance of the scar(s) | 23 (31.5%) | 23 (31.5%) | 25 (34.2%) | 2 (2.7%) |
| Scar feeling | 9 (12.3%) | 25 (34.2%) | 35 (47.9%) | 4 (5.5%) |
| Scar flexibility | 3 (4.1%) | 19 (26.0%) | 48 (65.8%) | 3 (4.1%) |
| Being able to cope with heat | 12 (16.4%) | 19 (26.0%) | 40 (54.8%) | 2 (2.7%) |
| Not being anxious | 22 (30.1%) | 12 (16.4%) | 33 (45.2%) | 6 (8.2%) |
| Not having nightmares | 24 (32.9%) | 16 (21.9%) | 24 (32.9%) | 9 (12.3%) |
| Feeling happy or cheerful | 14 (19.2%) | 17 (23.3%) | 37 (50.7%) | 5 (6.8%) |
| Having self-confidence | 14 (19.2%) | 19 (26.0%) | 35 (47.9%) | 5 (6.8%) |
| Not thinking back to the incident | 27 (37.0%) | 21 (28.8%) | 20 (27.4%) | 5 (6.8%) |
| Not having stress | 15 (20.8%) | 19 (26.4%) | 33 (45.8%) | 5 (6.9%) |
| Not feeling depressed | 17 (23.6%) | 16 (22.2%) | 33 (45.8%) | 6 (8.3%) |
| Not feeling guilty or ashamed | 27 (37.5%) | 16 (22.2%) | 23 (31.9%) | 6 (8.3%) |
| Trusting your body | 15 (20.8%) | 19 (26.4%) | 33 (45.8%) | 5 (6.9%) |
| Being able to think well | 21 (29.2%) | 8 (11.1%) | 38 (52.8%) | 5 (6.9%) |
| Walking or moving around | 6 (8.5%) | 9 (12.7%) | 47 (66.2%) | 9 (12.7%) |
| Lifting or moving something | 5 (7.0%) | 10 (14.1%) | 48 (67.6%) | 8 (11.3%) |
| Fine hand motor skills | 9 (12.7%) | 7 (9.9%) | 40 (56.3%) | 15 (21.1%) |
| Taking care of yourself | 9 (12.7%) | 7 (9.9%) | 45 (63.4%) | 10 (14.1%) |
| Doing household chores | 10 (14.1%) | 16 (22.5%) | 39 (54.9%) | 6 (8.5%) |
| Carrying out hobbies | 5 (7.0%) | 17 (23.9%) | 44 (62.0%) | 5 (7.0%) |
| Returning to work | 13 (18.3%) | 6 (8.5%) | 44 (62.0%) | 8 (11.3%) |
| Not having financial worries | 12 (16.9%) | 6 (8.5%) | 46 (64.8%) | 7 (9.9%) |
| Being independent | 6 (8.5%) | 13 (18.3%) | 47 (66.2%) | 5 (7.0%) |
| Your appearance | 19 (26.8%) | 17 (23.9%) | 30 (42.3%) | 5 (7.0%) |
| Interacting with people/strangers | 24 (33.8%) | 18 (25.4%) | 24 (33.8%) | 5 (7.0%) |
| Interacting with friends/colleagues | 17 (23.9%) | 15 (21.1%) | 34 (47.9%) | 5 (7.0%) |
| Interacting with your boss | 25 (35.2%) | 10 (14.1%) | 18 (25.4%) | 18 (25.4%) |
| Interacting with family | 15 (21.1%) | 9 (12.7%) | 42 (59.2%) | 5 (7.0%) |
| Interacting with partner | 13 (18.3%) | 13 (18.3%) | 33 (46.5%) | 12 (16.9%) |
| Physically being able to have sex | 13 (18.3%) | 15 (21.1%) | 32 (45.1%) | 11 (15.5%) |

**Appendix 4.** Top-10 most important short-term and long-term outcomes for patients without surgery, one surgery and two or more surgeries

**
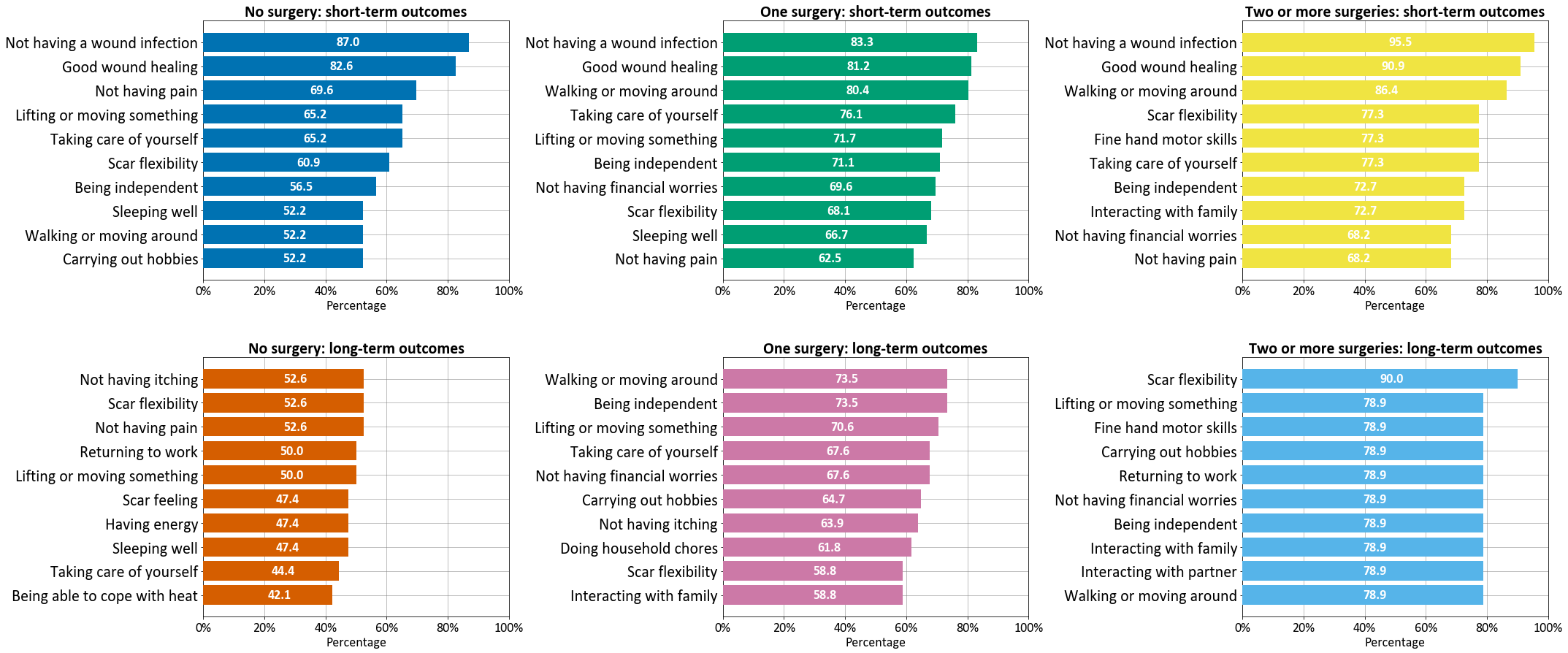
**
